# Coronavirus Disease 2019 Emergency and Remote Eye Movement Desensitization and Reprocessing Group Therapy With Adolescents and Young Adults: Overcoming Lockdown With the Butterfly Hug

**DOI:** 10.3389/fpsyg.2021.701381

**Published:** 2021-08-25

**Authors:** Elisa Lazzaroni, Roberta Invernizzi, Elisa Fogliato, Marco Pagani, Giada Maslovaric

**Affiliations:** ^1^Asst Lecco, Lecco, Italy; ^2^Institute of Cognitive Sciences and Technologies, Consiglio Nazionale delle Ricerche (CNR), Rome, Italy; ^3^Association EMDR Italy, Varedo, Italy

**Keywords:** COVID-19, lockdown, EMDR, telemedicine, adolescence

## Abstract

The coronavirus disease 2019 (COVID-19) pandemic has represented an individual and collective trauma with an impact on mental health. Restrictive measures such as lockdowns have increased risk factors for the development or triggering of various psychopathologies. Timely psychological intervention has constituted a protective factor that has been indicated as a form of prevention. The main objective of this study was to measure changes in the levels of traumatic stress and anxiety in a clinical population of adolescents and young adults aged 13 to 24 years – already assisted by the local primary and specialty care services before the pandemic – following a trauma-focused psychotherapeutic group intervention according to the eye movement desensitization and reprocessing protocol, conducted remotely before the end of the first lockdown. The Impact of Event Scale-Revised (IES-R), State-Trait Anxiety Inventory (STAI) scales, and the Emotion Thermometer were administered pre- and post-treatment. At the end of the treatment, the Post-Traumatic Growth Inventory (PTGI) questionnaire was administered. The results show that there was a significant improvement pre- and post-intervention in the scores of the scales STAI, IES-R, and Emotion Thermometer with a reduction in post-traumatic symptoms related in particular to the domains of intrusiveness and hyperarousal. The domain of avoidance was less significantly modified by therapy. This overall clinical improvement did not correlate with any of the demographic variables of the sample. In addition, the results show a significant positive global perceived change (PTGI) that did not correlate with the reduction of anxiety or post-traumatic symptoms measured by the other self-report scales. The explored use of telemedicine has revealed a valuable clinical opportunity.

## Introduction

On March 11, 2020, the WHO announced the first pandemic caused by a coronavirus. Many countries declared a state of emergency and took strict public health measures to prevent the spread of the virus, to the point of locking down cities (Ellis et al., 2020). In Italy, the lockdown for the coronavirus disease 2019 (COVID-19) pandemic began on March 9, 2020 and ended on May 18, 2020.

Physical isolation, in association with economic instability, fear of the spread of the infection, and uncertainty about the future, has had a profound impact on global mental health, imposing a reorganization of clinical activity (Invernizzi et al., 2020; Li et al., 2020) and defining new research horizons (Brooks et al., 2020; Holmes et al., 2020).

A survey on the mental health of the general population in China 2 weeks after the COVID-19 outbreak showed that about one-third of participants reported a moderate to severe level of anxiety (Wang G. et al., 2020) and about 40% of young people showed a tendency to present psychological problems (Liang et al., 2020a, p.1165). In particular, those who had shown higher levels of psychological distress, such as anxiety, depression, and fear, were more likely to develop symptoms of post-traumatic stress disorder (PTSD; Liang et al., 2020a, p. 1165; Wang W. et al., 2020; Xi et al., 2020). Lifestyle changes and fear of being infected caused anxiety and depressive disorders (Chen et al., 2020). Similar results had already been found in studies conducted on individuals who had been quarantined following the 2003 severe acute respiratory syndrome (SARS) epidemic. These subjects presented a much higher prevalence of symptoms of anxiety, stress, depression, irritability, insomnia, and post-traumatic symptoms than the non-quarantined subjects (Brooks et al., 2020).

This study aimed at investigating how the traumatic nature of the pandemic impacted on adolescents and young adults of a clinical population as individuals and as a community, considering that their developmental phase involves, by its nature, a strong investment in formative tasks related to the interaction with peers and future planning (Oosterhoff et al., 2020).

What happened to young people living in an emergency situation in which they had to face a lockdown condition? Adolescents and young adults forced to stay at home, attend school remotely, and observe physical and social distancing represent a group at risk of experiencing even a greater stressful impact than the rest of the population (Findlay et al., 2020). In particular, some studies have shown that adolescent boys appear to have suffered more from social distancing (Buzzi et al., 2020), while adolescent girls have presented more symptoms attributable to PTSD (Liang et al., 2020b) and a higher risk for depression and anxiety (Chen et al., 2020). In fact, all were exposed to a trauma that could block the capability of planning for and envisioning the future, and some expressed the belief that they were now too far behind with the phase-specific growth tasks of their age (Navarra, 2020). On the contrary, in the face of a collapse of hope, adolescents and young adults have also shown that they know how to reorganize themselves, moving online, to the virtual world, everything they could, from classes to meetings with friends, from work to leisure activities, slipping into a new routine that has helped them to cope with physical distancing also in its social valence (Navarra, 2020). Recent studies have shown that adolescents seem to have a better ability to cope with the adverse living conditions experienced during the COVID-19 pandemic than the adult population (Buzzi et al., 2020).

Other studies have demonstrated that pandemic-related stress levels in these young people appear to be associated with heightened levels of depression and loneliness (Chen et al., 2020; Ellis et al., 2020), also linked to staying home alone during the week. In contrast, time spent with family members, virtual connection with friends, and physical activity appears to be protective factors (Ellis et al., 2020) for mental health, as well as access to psychological support and school rescheduling (Sharma et al., 2020). Indeed, without appropriate psychological interventions, depression and anxiety among adolescents can also become risk factors for mental disorders in adulthood (Danese et al., 2009; Jones, 2013), and it is the promptness of intervention that reduces the prevalence of PTSD (Zhou et al., 2013). In this regard, it is important to emphasize that traumatic events lead those exposed to them to experience feelings of helplessness, hypervigilance, and alarm and experience negative emotions that predispose to negative coping patterns, which are more likely to make psychological distress evolve into full-blown PTSD (Vlahov et al., 2002). Adolescents, who tend to experience emotions with higher intensity due to their specific stage of development (Sahoo et al., 2020), may be at greater risk in this perspective.

Isolation, reduced physical activity, instability of the situation, fear of infection, or the presence of sick relatives in the family may also increase the risk factors for depression, anxiety, post-traumatic disorders, and suicidality, particularly in already vulnerable adolescent populations (Hao et al., 2020; Szlyk et al., 2020) such as those with symptomatic medical histories. Studies confirm the severity of the negative psychological impact of strict lockdown measures on psychiatric patients during the pandemic (Hao et al., 2020).

With the COVID-19 outbreak, the need for evidence-based online PTSD treatments has proved to be urgent. In particular, social distancing measures that have been implemented in many countries to reduce the spread of COVID-19 have forced clinicians to deliver treatment *via* audio/video call, e-mail, or the Internet. Continuing distant-delivered treatment during the pandemic is pivotal because psychiatric patients seem more vulnerable to experience worsening of symptoms after the COVID-19 outbreak compared with people without psychiatric complaints (Hao et al., 2020; Lenferink et al., 2020). Even in our local services for adolescents and young adults, it was essential, during the lockdown, to continue delivering remotely the psychological interventions that were already underway, not only to ensure continuity of care but also to prevent, in the second place, the onset of pandemic-related disorders in the area of mental health (Barney et al., 2020).

The clinical study population was formed by a group of adolescents and young adults aged between 13 and 24 years already assisted by the local primary and specialty care services of the Azienda Socio Sanitaria Territoriale (Asst) of Lecco (Northern Italy) that presented developmental risk factors related to the exposure to collective traumatic events, such as the pandemic. Therefore, attention was paid to the peri- and post-traumatic symptomatology linked to the experience of the coronavirus during the first lockdown in Italy (February–May 2020), proposing a trauma-focused psychotherapeutic group intervention with eye movement desensitization and reprocessing (EMDR) delivered remotely due to the measures imposed by the COVID-19 pandemic. Post-treatment changes in the levels of traumatic stress and anxiety were measured, and it was assessed whether there had been any personal and interpersonal growth opportunities related to the pandemic context experienced in the first emergency wave. This treatment was proposed before the end of the lockdown (May 2020) in a perspective of prevention, for an adaptive return to life and social relationships in an already vulnerable population of adolescents and young adults.

In summary, the main focus of the study is to detect, in an evolutive and prevention perspective, peri- and post-traumatic symptomatology linked to the experience of the coronavirus during the first lockdown in Italy measured before and after a trauma-focused psychotherapeutic group intervention with EMDR delivered remotely. We expect to discuss the following hypotheses:

- The experience of coronavirus during the first lockdown has had a strong impact on the mental health of adolescents and young adults in terms of post-traumatic stress.
- The EMDR group intervention delivered remotely during the first emergency wave to assist a population of adolescents and young adults, already clinically vulnerable, in the exit from the first lockdown has a positive effect in reducing anxiety and post-traumatic symptoms.
- The pandemic experience could cause personal growth and interpersonal growth that result as the tendency, following a trauma, to report positive changes in three main areas: change in self-perception, interpersonal relationships, and philosophy of life.
- The treatment proposed before the end of the lockdown (May 2020) is a helpful intervention in the perspective of prevention and for an adaptive return to life and social relationships in an already vulnerable population of adolescents and young adults.
- The use of telemedicine in emergencies could be a valuable opportunity.

## Materials and Methods

### Setting

Adolescents and young adults were recruited in two settings of the Asst of Lecco: a psychological service dedicated to young people aged between 15 and 24 years (project #quindiciventiquattro) with common emotional disorders and a local Child and Adolescent Neuropsychiatry service dedicated to adolescents aged between 13 and 18 years.

Participation took place in compliance with the privacy and protection of the sensitive data of those involved, as per company forms. The meetings were held online in compliance with the regulations in force issued by the government during the COVID-19 emergency phase.

### Subjects

We recruited 50 adolescents and young adults assisted by the local healthcare services prior to the pandemic who continued to receive psychological care remotely during the lockdown.

Inclusion criteria were as follows: (1) psychological care already initiated, (2) age between 13 and 24 years, and (3) willingness to initiate group treatment focused on COVID-19 trauma. We only involved stabilized clinical cases.

The exclusion criteria were as follows: (1) presence of severe psychopathological conditions (in particular, we excluded subjects with schizophrenia, severe psychosis, delirious disorders, and psychotic onsets, and we also excluded subjects with not yet stabilized clinical frames, for example, patients who have had access to the emergency room or been hospitalized in the past 6 months), (2) suicidal ideation or suicide attempts, and (3) cognitive deficits. For all these patients, a trauma-focused COVID-related group and online treatment were not the clinical priority, and the setting could not be appropriate.

Due to the logistic problems and to avoid overloading the adolescents with excessive tests, the SCL-90 was not administered (see the “Limitations” section of the study). The characteristics of the population are described in Table 1.

**TABLE 1:** Demographic variables (n%).

**Gender**	

F	84%
M	16%

**Education**	

Elementary school	6%
Bachelor’s Degree	4%
Junior high school	82%
High school	8%

**Relatives that died from Covid-19**	

NO	94%
YES	6%

**Domicile during the pandemic**	

Isolated in the family (family member in quarantine at home)	14%
Not isolated in the family (nobody quarantined)	86%

**Relatives that tested positive for Covid-19**	

NO	80%
YES	20%

**Diagnosis**	

F2	4%
F3	22%
F4	44%
F5	10%
F6	10%
F9	10%

### Assessment

Recruitment of the adolescents and young adults was managed by the therapist who conducted the individual psychological sessions remotely with them during the lockdown. EMDR group treatment focused on the COVID-19-related trauma and the aim of offering support when coming out of lockdown were explained. The use of the TEAMS platform for remote meetings was proposed and shared. Participants were divided into small groups of three or four people. The therapist who facilitated the group was qualified to use EMDR. All EMDR therapists were supervised by an EMDR consultant.

For each subject, a personal data sheet was compiled, which included some COVID-19-related life events such as the presence of family members who had been infected or had died from COVID-19, and the isolation of a sick family member at home due to quarantine.

The following self-report scales were administered before and after the group intervention:

*Impact of Event Scale-Revised (IES-R*): This scale was used to measure stress levels and symptomatology due to the impact of the traumatic event of the pandemic. The IES-R (Weiss and Marmar, 1997) is a 22-item self-report questionnaire consisting of three subscales (eight items relate to intrusions, eight items evaluate avoidance, and six items assess hyperarousal). The scale assesses the subjective distress caused by traumatic events. Participants were asked to rate each item on a scale from 0 (not at all) to 4 (extremely), based on their experience with the traumatic event in the previous 7 days. An IES-R score ≥ 33 represents the best cut-off for a probable diagnosis of PTSD. The IES-R was found to be highly internally consistent (Cronbach’s alpha, α = 0.96; Creamer et al., 2003).

*StateTrait Anxiety Inventory* (*STAI-Y*): This scale was designed by Spielberger et al. (1983), in the Italian version contained in the CBA 2.0 battery by Cilia and Sica (1998), with the aim of measuring the levels of state anxiety through the STAI-Y1 subscale. The STAI-Y1 (Spielberger et al., 1983) is used to measure the presence and severity of current symptoms of anxiety (state anxiety). The subject rates on a scale of 1 to 4 (with 1 = not at all and 4 = very much) how well various statements fit his or her behavior. Range of scores for each subtest is 20–80, the higher score indicating greater anxiety. A cut point of 39–40 has been suggested to detect clinically significant symptoms for the state anxiety scale. The STAI-Y has shown an adequate to excellent internal reliability (Cronbach’s alpha, α = 0.86–0.95).

*Emotion Thermometer* (Mitchell et al., 2010): This is a visual analog self-assessment scale used to measure the level of intensity of emotional activation on a Likert scale from 1 to 10 regarding some main emotional experiences (e.g., stress, depressed mood, anxiety, anger, sleep problems, and need for help) during the previous week.

At the end of the intervention, the following was also administered:

*Post-Traumatic Growth Inventory* (*PTGI*; Tedeschi and Calhoun, 1996; Prati and Pietrantoni, 2014): This is a self-report questionnaire on post-traumatic growth used to measure any personal and interpersonal changes related to the pandemic event. The scale consists of 21 items with response mode on a Likert scale from 0 (no change) to 5 (very important change) and measures the positive outcomes reported by people that faced negative and adverse experiences (Cormio et al., 2017). The total score ranges from 0 to 105. The PTGI was found to be highly internally consistent (Cronbach’s alpha, α = 0.92; Prati and Pietrantoni, 2006). Post-traumatic growth is defined as the tendency, following a trauma, to report positive changes in three main areas: change in self-perception, interpersonal relationships, and philosophy of life (Tedeschi et al., 1998). Tedeschi and Calhoun (1996) identified five dimensions on which post-traumatic growth acts and on which the PTGI scale is based: (i) a social dimension, which refers to closeness with others; (ii) a cognitive dimension, which refers to feeling stronger and more capable of solving problems; (iii) an emotional dimension, which includes greater compassion for the suffering of others and better expression of emotions and feelings; (iv) a physical dimension, like assuming a healthier lifestyle; and (v) finally, a spiritual dimension, which refers to changing priorities in life. The underlying construct is the theory that people who have undergone traumatic experiences and struggled with highly demanding and challenging life circumstances can then experience a positive change within themselves, such as developing a new appreciation for life, a newfound sense of personal strength, or a new focus on helping others (Collier, 2016).

### Treatment

#### Eye Movement Desensitization and Reprocessing

The subjects of the study participated in three group meetings of 1 h each, delivered online (in compliance with the rules and authorizations provided by the protocols of telemedicine) according to the *brief EMDR group treatment* protocol created by the re-elaboration of the guidelines for the stabilization-decompression of Critical Incident Stress Management (CISM, Everly et al., 2001; Quinn, 2009) and the specific EMDR protocols for Acute and Recent Traumatic Events (Shapiro and Laub, 2008). For alternating bilateral stimulation, the butterfly hug was used (Maslovaric, 2020).

Eye Movement Desensitization and Reprocessing (EMDR) is a therapeutic approach used for the treatment of trauma and traumatic stress-related issues (Shapiro, 2000) based on the adaptive information processing (AIP) model (Shapiro, 2000). According to the AIP, the traumatic event experienced by the subject is stored in memory together with the disturbing emotions, perceptions, cognitions, and physical sensations that characterized that moment. All the information stored in a dysfunctional way remains “frozen” within the neural networks and cannot connect to other networks with useful data (Fernandez and Giovannozzi, 2012); unable to be processed, it continues to cause discomfort in the subject, up to the onset of diseases such as PTSD and other psychological disorders. The aim of EMDR is to restore the natural way of processing the information in the memory to achieve an adaptive resolution through the creation of new, more functional connections. A distinct characteristic of EMDR therapy is the use of alternating bilateral stimulation (e.g., eye movements, tactile stimulation, auditory stimulation, and butterfly hug), which appears to produce a physiological effect promoting accelerated reprocessing of dysfunctionally stored information related to the traumatic event (Jeffries and Davis, 2013; Carletto et al., 2017; Pagani et al., 2017). EMDR is considered as one of the elective psychotherapeutic treatments for PTSD, according to several meta-analyses and clinical guidelines, and its neurobiological effects are also supported by neuroimaging findings (Pagani et al., 2012; Carletto et al., 2018, p. 2).

At present, it is recognized as an evidence-based method for the treatment of post-traumatic disorders (Baek et al., 2019; Maddox et al., 2019) approved by the *American Psychological Association* (1998–2002), the *American Psychiatric Association* (2004), the *International Society for Traumatic Stress Studies* (2010), and the *Italian Ministry of Health* in 2003. The WHO in August 2013 recognized EMDR as an effective treatment for trauma and trauma-related disorders.

### Statistical Methods

#### Preliminary Coding

The data relating to the 50 subjects were preliminary coded to facilitate the analysis and interpretation of results. This coding, while keeping invariant the original meaning of the variables, allowed for a less biased statistical treatment. This process involved the following:

1. Generation of an ordinal variable named rankscol corresponding to the educational level of the subjects, thus elementary school = 1, junior high school = 2, high school = 3, and bachelor’s degree = 4. The natural ordering of the different school grades allows considering only one rank variable instead of four qualitative ones.
2. Elimination of the variables with null (or almost null) variance in the dataset. As a matter of fact, a variable with all identical results does not carry any relevant information (i.e., this was the case of the variable “tested positive for COVID-19” that has all negative answers).
3. Merging less populated classes. This allows to get a more sensible way to perform chi-square statistics or other nominal variables analyses. This merging kept invariant the content of the relative variable for the lockdown variable that was reduced to two entries no quarantine/quarantine for no relatives isolated and one or more quarantined relatives, respectively.

In order to autoscale both the IES and Emotion Thermometer variables, the respective items (each one computed in the pre- and post-conditions) were expressed in terms of differential values: delta = pre–post. All the subsequent correlation analyses were executed on such delta variables.

#### Emerging Properties and Chance Correlation Reduction

In order to both reduce the risk of chance correlations and evaluate the presence of emergent collective properties from single items, we collapsed all the test variables into only one integrative index correspondent to the first principal component of IES and thermometer items (deltaf1), represented in delta terms (see Factor procedure in SAS 11.2 software earlier). This was made possible by the very strong mutual correlation among the different original variables and endowed with all positive loadings (correlation coefficients between original variables and component). This made deltaf1 a “size” component (Jolicoeur and Mosimann, 1960) directly registering the coherent entity of change of all the studied parameters. Principal component analysis (PCA) was computed over the correlation matrix of the original variables so giving rise to normalized (*Z*-scores) component scores having zero mean and unit standard deviation on the entire set of subjects. This means that a subject with a negative component score has a “less than average” amelioration, while a subject with a positive component score has a “greater than average” response.

We adopted the same procedure for PTGI data. In this case, the main variabile (changeglobal) explained 64% of total variance collecting all the items with the only exception of “spiritual change” that generated an independent component of its own (PC2) we named spiritual change accounting for 17% of variance. This component in any case is largely irrelevant given only a minority of subjects scored a different from 0 delta as for spiritual change.

#### Inferential Statistics

The influence of demographic nominal variables on both deltaf1 and changeglobal was assessed by ANOVA (General Linear Model [GLM] procedure in SAS 11.2 software), while Spearman’s correlation coefficient (CORR procedure in SAS 11.2 software) was applied to check the influence of ordinal and quantitative variables on the test variables.

When submitted to paired *t*-test, almost all the variables showed a statistically significant departure from delta = 0, corresponding to the no-change condition.

## Results

First (Table 2), we extracted the main principal components from the original 11 variables dataset corresponding to the IES and thermometer items. The five-component solution explained around 82% of total variance with the first component (deltaf1) accounting alone for 40% of the original information. As mentioned in the Methods section, deltaf1 emerges as a global index upon which the effect of covariates is tested.

**TABLE 2:** PCA on delta variables (IES and Thermometer).

	Deltaf1	Deltaf2	Deltaf3	Deltaf4	Deltaf5
Deltaiesavoidance	**0.59**	–0.45	–0.36	0.07	0.27
Deltaiesintrus	**0.70**	–0.44	0.36	–0.09	–0.05
deltaieshypera	**0.78**	–0.40	–0.03	–0.09	–0.14
Deltaiestotal	**0.84**	–0.53	–0.01	–0.04	0.04
delta stress	**0.62**	0.50	–0.24	–0.32	–0.20
delta anxiety	**0.60**	0.47	0.16	–0.17	–0.22
delta mood	**0.61**	0.51	–0.13	0.32	0.10
delta anger	**0.73**	0.16	–0.28	–0.16	–0.18
delta sleep	0.40	0.02	0.31	**0.77**	–0.31
delta help	0.34	0.22	**0.75**	–0.28	0.27
Deltastaix	**0.58**	0.38	–0.09	0.23	0.53

*In bold the variables grouped in the same component.*

All single IES-R and thermometer variables showed post-EMDR highly significant changes in delta scores albeit with differences between one and the other (Table 3).

**TABLE 3:** Univariate statistics relative to the delta variables.

Variables	Mean	Standard deviation	*P*<
deltaiesavoidance	2.72	5.05	**0.0005**
Deltaiesintrus	4.04	5.22	**0.0001**
deltaieshypera	3.94	4.37	**0.0001**
Deltaiestotal	10.70	11.90	**0.0001**
delta stress	2.00	2.72	**0.0001**
delta anxiety	2.00	2.71	**0.0001**
delta mood	1.22	2.65	**0.005**
delta anger	0.86	2.90	**0.05**
delta sleep	0.76	2.69	**0.05**
delta help	1.54	2.54	**0.0001**

*In bold the statistical significances.*

As evident from Table 4, deltaf1 has no statistically significant correlation with demographic variables, with the exception of the correlation between age and rankscol. The latter is largely trivial, being linked to the progression of students along with the school curriculum.

**TABLE 4:** Spearman correlation matrix and *p* values between deltaf1 and demographic variables.

	Age	rankscol	n. of live-in members	DELTAF1
Age	100.00	0.69	0.013	–0.02
		** < 0.0001**	0.93	–0.87
Rankscol	0.69	100.00	0.08	–0.02
	** < 0.0001**		0.57	0.89
n. of live-in members	0.01	0.08	100.00	0.02
	0.93	0.57		0.87
DELTAF1	–0.02	–0.02	0.02	100.00
	0.87	0.89	0.87	

*In bold the statistical significances.*

Table 5 reports the PCA relative to PTGI variables; as anticipated in the Methods section, even here we have a first principal component collecting the major portion of variance (64%) and practically coincident with the delta between the sum over all items (Pearson’s *r* between changeglob and PTGI sum = 0.99).

**TABLE 5:** PCA relative to PTGI variables.

	changeglobal	changespirit	PC3
relatingtoothers	**0.85**	–0.19	0.07
newpossibilities	**0.92**	–0.03	0.02
Personalstrength	**0.83**	–0.30	0.35
spiritualchange	0.50	**0.85**	0.15
appreciationoflife	**0.83**	0.01	–0.52

*In bold the variables grouped in the same component.*

Table 6 reports the Spearman’s correlation between PTGI components and demographic numerical and ordinal variables. Beyond age, there are two weak albeit statistically significant results, pointing to a smaller change for subjects living in big families (Spearman’s correlation between changeglob and number of live-in members = −0.33) and a more marked spiritual change in lower degree students (*r* = −0.30).

**TABLE 6:** Spearman correlation matrix and *p* values between PTGI components and demographic numerical and ordinal variables.

	Changeglob	changespirit	age	rankscol	n. of live-in members
Changeglob	100.00	–0.19	–0.15	–0.05	–0.33
		0.18	0.29	0.71	**0.02**
changespirit	–0.19	100.00	–0.20	–0.30	0.02
	0.18		0.17	**0.03**	0.90
Age	–0.15	–0.20	100.00	0.69	0.01
	0.29	0.17		** < 0.0001**	0.93
Rankscol	–0.05	–0.30	0.69	100.00	0.08
	0.70	**0.03**	**< 0.0001**		0.57
n. of live-in members	–0.33	0.02	0.01	0.08	100.00
	**0.02**	0.90	0.93	0.57	

*In bold the statistical significances.*

It is worth noting that PTGI and deltaf1 variables are independent of each other, so pointing to a different dimension of change with respect to emotional and psychological parameters registered by PTGI variables (Table 7).

**TABLE 7:** Pearson correlation coefficients relative to main principal components of IES, Thermometer and PTGI.

	DELTAF1	changeglob	changespirit
DELTAF1	100.00	–0.14	–0.02
		0.35	0.88
changeglob	–0.14	100.00	0.00
	0.35		10.00
changespirit	–0.022	0.00	100.00
	0.88	10.00	

The PTGI scored 15.88 ± 7.74 for relating to others, 10.82 ± 6.1 for new possibilities, 9.86 ± 4.93 for personal strength, 0.72 ± 1.26 for spiritual change, and 7.48 ± 3.48 for appreciation of life.

As for STAI-Y, the delta between pre- and post-treatment had a mean of 5.08 ± 10.56 with a significance level of *p* < 0.002.

## Discussion

The analysis by principal components revealed a first size component (deltaf1) which, representing 40% of the variance, grouped together the differential values of most of the variables in question. Specifically, there was a significant pre- and post-treatment improvement in the scores of the STAI-Y1, IES-R, and Emotion Thermometer scales. The results point to a post-intervention reduction in anxiety levels and post-traumatic symptomatology in line with previous studies (de Roos et al., 2011; Chen et al., 2014; Wilson et al., 2018; Yunitri et al., 2020). Although the current health emergency is unprecedented, these findings confirm that early intervention with EMDR has positive effects on reducing psychological distress, as it was found in populations exposed to natural disasters such as earthquakes (Saltini et al., 2018).

Regarding the change in the level of psychological distress measured by IES-R, there was a higher reduction of symptoms related to the domains of intrusiveness and hyperarousal. This finding confirms the effectiveness of EMDR in reducing the emotional impact of the stressful event linked to COVID-19 and the lockdown: intrusive thoughts are mitigated, becoming more adaptive from a therapeutic point of view, and emotions and physical sensations are reduced in intensity. The decrease in hyperarousal suggests a higher capacity to manage anxiety, functional to the possibility of more adaptive behaviors when the lockdown ends. In line with these findings, we can also read the reduction of the overall scores on the STAI-Y1: youngsters perceive post-intervention lower levels of anxiety and stronger feelings of calm and safety.

The improvement in the domain of avoidance is statistically significant but to a lesser extent. The data confirm what emerged from other studies (Saltini et al., 2018): the aspects of avoidance persist more over time within the mental functioning of the individual. With reference to our population and considering that the COVID-19 pandemic is occurring in several emergency waves, the persistence of aspects related to avoidance could have an adaptive nature, functional to the fact that phase-specific growth tasks like the capacity of planning for and envisioning the future are hindered by the lockdown.

The deltaf1 component does not comprise the measurement of sleep disturbance and the need for help included in the Emotion Thermometer, which remain stable over time. It is presumed that, in the first case, the absence of pre- and post-treatment change is due to the condition of being hyper-connected, amplified by distance learning during the lockdown; and in the second case, to the fact that all subjects were in therapy even before the outbreak of the pandemic.

The overall clinical improvement does not correlate with any of the demographic variables of the sample taken into consideration, from which it appears independent. Nor does it correlate with the level of post-traumatic growth, as shown also in other studies (Jeon et al., 2017).

With respect to post-traumatic growth, the results confirm a perceived global positive change (changeglobal at PTGI). It should be considered the limitation related to the questionnaire, which records only positive changes and not negative ones.

Finally, it can be speculated that the overall clinical improvement and the global post-intervention perceived positive changes indicate the preventive function that early treatment with EMDR may have had on the exit from lockdown as a COVID-19-related life event.

The significant modifications recorded pre- and post-remotely delivered EMDR group treatment are in line with previous studies conducted face to face (Chen et al., 2014; Saltini et al., 2018; Wilson et al., 2018), confirming the importance of telemedicine in emergencies. However, further studies are needed to evaluate the effects of face-to-face vs. online treatments, as well as face-to-face EMDR vs. guided online EMDR (Lenferink et al., 2020).

An interesting prospect will be to be able to monitor changes over time with respect to new waves of emergency by comparing the scores of the sample with the scores of adolescents and young adults assisted by the local healthcare services but not treated with EMDR.

### Limitations

The main limitation is the sample size, especially when compared with the number of items administered with the scales. Being aware of the possible flaw, as for the IES-R (22 items) and PTGI (21 items), we used only four and five cumulative scales, deriving from the summation of specific subsets of items, respectively, and only one cumulative value for the STAI-Y (20 items).

Furthermore, to overcome the above-sketched limitation, we performed the PCA on the delta values of IES-R and thermometer items demonstrating the presence of five components explaining 82% of the variance (40% for the first component only). The emergence of such a global index corroborated the effect of treatment on the single variables. It has to be stressed that all items gave a significant result in terms of pre- and post-treatment of delta variables.

Another limitation Is represented by the absence of a self report scale to detect psychopathological diagnoses of any instrument for general psychopathology; to avoid overloading the adolescents with excessive tests, the SCL-90 was not administered.

### Conclusion

The COVID-19 pandemic was and is a traumatic personal and collective event that occurs in multiple emergency waves, imposing repeated lockdowns. The EMDR group intervention delivered remotely during the first emergency wave to assist a population of adolescents and young adults, already clinically vulnerable, in the exit from the first lockdown had a positive effect in reducing anxiety and post-traumatic symptoms and was accompanied by a perception of global positive change. The need imposed by the pandemic to explore new frontiers as therapists, particularly the use of telemedicine in emergencies, proved to be a valuable opportunity. Therefore, the significant change before and after online group treatment with EMDR as measured through the self-report scales encourages us to continue in this direction, despite all the uncertainties we have experienced in this pandemic, both as professionals and as adults, in order to rewrite the hope in a possible future with the adolescents and young adults assisted by the local healthcare services.

## Data Availability Statement

The raw data supporting the conclusions of this article will be made available by the authors, without undue reservation.

## Ethics Statement

Ethical review and approval was not required for the study on human participants in accordance with the local legislation and institutional requirements. Written informed consent to participate in this study was provided by the participants’ legal guardian/next of kin.

## Author Contributions

RI, EF, and GM contributed to conception and design of the study. RI and EF organized the database. MP performed the statistical analysis. EL, RI, and EF wrote the first draft of the manuscript. All authors wrote sections of the manuscript, contributed to manuscript revision, read, and approved the submitted version.

## Conflict of Interest

The authors declare that the research was conducted in the absence of any commercial or financial relationships that could be construed as a potential conflict of interest.

## Publisher’s Note

All claims expressed in this article are solely those of the authors and do not necessarily represent those of their affiliated organizations, or those of the publisher, the editors and the reviewers. Any product that may be evaluated in this article, or claim that may be made by its manufacturer, is not guaranteed or endorsed by the publisher.
